# Short-term intensive psychodynamic group therapy versus cognitive-behavioral group therapy in day treatment of anxiety disorders and comorbid depressive or personality disorders: study protocol for a randomized controlled trial

**DOI:** 10.1186/s13063-015-0827-6

**Published:** 2015-07-29

**Authors:** Hubert Suszek, Paweł Holas, Tomasz Wyrzykowski, Steinar Lorentzen, Andrzej Kokoszka

**Affiliations:** Faculty of Psychology, University of Warsaw, Ul. Stawki 5/7, 00-183 Warsaw, Poland; Wola Hospital, ul. Kasprzaka 17, 01-211 Warsaw, Poland; Institute of Clinical Medicine, University of Oslo, PO Box 1039, , Blindern, Oslo, 0315 Norway; II Department of Psychiatry, Medical University of Warsaw, ul. Kondratowicza 8, 03-242 Warsaw, Poland

**Keywords:** Anxiety disorders, Depressive disorders, Personality disorders, Psychodynamic psychotherapy, Cognitive-behavioral psychotherapy, Group psychotherapy, Day treatment

## Abstract

**Background:**

Psychodynamic and cognitive-behavioral group therapies are frequently applied in day hospitals for the treatment of anxiety disorders and comorbid depressive or personality disorders in Poland and other Eastern European countries. Yet there is not enough evidence as to their effectiveness in this environment; this study addresses this gap. The aim of the study is to determine the effectiveness of these two kinds of day treatment care consisting of intensive, short-term group psychodynamic and cognitive-behavioral therapy, for patients with anxiety disorders and/or comorbid depressive or personality disorders. Our objectives are to: 1) show the effectiveness of each treatment in a day-care setting relative to the wait-list control group; 2) demonstrate the relative short- and long-term effectiveness of the two active treatments; 3) carry out a preliminary examination of the predictors and moderators of treatment response; 4) carry out a preliminary examination of the mediators of therapeutic change; and 5) compare the impact of both methods of treatment on the outcome of the measures used in this study.

**Methods/Design:**

In this randomized controlled trial, a total of 199 patients with anxiety disorders and comorbid depressive and/or personality disorders will be assigned to one of three conditions: 1) psychodynamic group therapy; 2) cognitive-behavioral group therapy; or 3) wait-list control group. The therapy will last 12 weeks. Both treatments will be manualized (the manuals will address comorbidity). Primary outcome measures will include self-reported symptoms of anxiety, observer-rated symptoms of anxiety, global improvement, and recovery rate. Secondary outcome measures will include the number of pathological personality traits, depression, self-esteem, defense mechanisms, beliefs about self and others, interpersonal problems, object relations, parental bonding, meta-cognition, and quality of life. Measures will be taken at baseline, post-treatment, and at six months following the end of therapy.

**Discussion:**

The rationale is to investigate how effectively anxiety disorders and/or comorbid depressive or personality disorders can be treated in a day hospital setting, typical of the Polish health care system, during a three-month treatment period.

**Trial registration:**

Clinicaltrials.gov identifier NCT02126787, registered on 28 April 2014.

## Background

Group psychotherapy of severe neurotic disorders in a day unit setting has been widely developed in Eastern European countries since the 1960s. This was offered for patients who, due to the disabling intensity of their symptoms, could not function normally and therefore had poor quality of life, and were admitted for three months intensive treatment in day clinic settings. It usually took place five days per week for five to seven hours each day, and took the form of group psychotherapy [57]. This intensive short-term format is still the most available type of psychotherapy, reimbursed by the National Health Service in Poland, where 300 such wards exist today. Every year, approximately 8,000 individuals suffering from anxiety and/or personality disorders are treated in this setting in day clinics in Poland [38]. In spite of its popularity, the effectiveness of psychodynamic and cognitive-behavioral 12-week intensive group psychotherapy treatment for anxiety disorders in a day clinic setting has never been tested or compared with current methodological scrutiny, which has prompted the current research project.

Anxiety disorders are among the most prevalent mental disorders, with a lifetime prevalence of 30 % in the US [42] and 9.9 % in Poland [52], according to a study that used a similar methodology to that conducted in the US. Many studies suggest that in some countries there is a strong comorbidity with personality disorders. A recent meta-analysis of 125 studies [27] demonstrated that the risk of comorbid anxiety disorders and personality disorders varied from 35 to 52 %; the most frequently comorbid disorders are Cluster C personality disorders, as classified by the *Diagnostic and Statistical Manual of Mental Disorders* (DSM-V). Epidemiological studies also show high comorbidity rates for anxiety and depressive disorders, ranging from 40 to 80 % [39, 46]. Data on relationships between comorbid mental disorders and treatment outcomes are inconsistent. Reviews by Reich and Green [64] and Reich and Vasile [65] including 38 studies have shown that comorbid anxiety disorders and personality disorders are negatively related to treatment outcome. However, after analyzing 15 studies, Dreessen and Arntz [21] did not share these conclusions.

It should be noted that the comorbidity of anxiety disorders and personality disorders in the population of neurotic patients treated in day clinics seems to be higher than that reported in other studies. The results of one of our studies show that 125 out of 152 participants (82.3 %) fulfilled the criteria for at least one personality disorder (as assessed by the Structured Clinical Interview for DSM-IV Personality Disorders Questionnaire - II (SCID-II) [76]. The effectiveness of day care has been investigated to a very small degree, especially with regards to its frequent usage [50]. However, difficulties in identifying the basic effective components of the treatment, and the large diversity of the treatment programs make it difficult to generalize the results.

Among the small number of published studies on the effectiveness of psychotherapy in a day care setting, only four were randomized controlled trials (RCTs). Three of those studies compared psychotherapy in day care units to outpatient psychotherapy [2, 19, 80], and one study used a wait-list control group [63]. Tyrer *et al*. [80] found no significant differences between day care and outpatient care for anxiety disorders. Dick *et al*. [19] found a significant difference in improvement of personality disorder pathology in favor of day care. In the first study, the psychotherapy modality was not mentioned, and in the second, eclectic psychotherapy was applied. In another study by Arnevik *et al*. [2], day care treatment (18-week group psychodynamic and cognitive-behavioral psychotherapy) was compared to individual psychotherapy in patients with personality disorders; no significant differences in improvement were observed between the two groups. Piper *et al*. [63] found that day care patients suffering from affective disorders and personality disorders experienced a significant improvement that continued during follow-up after eight months (18-week group psychodynamic psychotherapy), as compared to patients in the wait-list control group. The authors of a Cochrane review of day care versus outpatient care noticed that because of the small number of existing studies, ‘there is only limited evidence to justify the provision of day treatment programs and transitional day hospital care, and no evidence to support the provision of day care centers’ [50]. Existing data are therefore not sufficient enough to justify the use of group psychotherapy in day care, and more high-quality research is needed. The question as to which treatment of the two most widely used forms of therapy, psychodynamic or cognitive-behavioral, is more effective also awaits an answer. These questions are relevant in light of the large expenditures incurred by the national health funds that finance the treatment. They are also important to the issue of health care in Poland, where this type of treatment is very common.

### Objectives

The main objective of the planned research is to evaluate the clinical effectiveness of short-term, intensive, psychodynamic, and cognitive-behavioral group psychotherapy for anxiety disorders and comorbid depressive or personality disorders in day care conditions.

The more specific objectives are to: 1) show the effectiveness of each treatment in a day care setting relative to the wait-list control group; 2) demonstrate the relative short- and long-term effectiveness of the two active treatments; 3) carry out a preliminary examination of the predictors and moderators of treatment response; 4) carry out a preliminary examination of the mediators of therapeutic change; and 5) compare the impact of both methods of treatment on the outcome of the measures used in this study.

## Methods/Design

### Study design

**Fig. 1:**
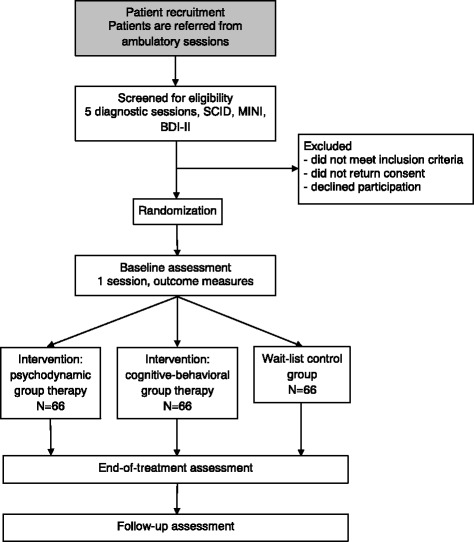
Flowchart of the trial design

This study is an RCT in which participants will be allocated to one of three conditions: 1) psychodynamic group therapy; 2) cognitive-behavioral group therapy; or 3) the wait-list control group (WL). Participants allocated to the wait list will commence treatment after a 12-week waiting period. The total duration of the study will be three years. Figure 1 shows the trial design. The study protocol, information brochure, and informed consent were approved by the Medical Ethics Committee of Warsaw Medical University (reference number: KB/61/2010).

### Study center

Both the protocol and the design comprise a part of the research grant given to the II Department of Psychiatry at the Medical University of Warsaw. The study will be carried out at the day unit of the Wola Center for Mental Health at Wola Hospital in Warsaw, which will be used as the clinical setting for the II Department of Psychiatry of the Medical University of Warsaw.

### Participant recruitment, inclusion and exclusion criteria and randomization

Participants with anxiety disorders and comorbid personality disorders will be recruited from an outpatient clinic. Participation is voluntary. Informed consent is given in writing and signed with a personal signature. Participants may withdraw their informed consent at any time, without any consequences for their treatment. Randomization will take place after informed consent has been obtained. Subjects who meet all of the inclusion criteria will be randomized to two modalities of treatments or to the wait list.

Patients from the wait list will continue pharmacological treatment and will take part in monthly supportive consultations. Crisis intervention will be offered when needed. In accordance with the informed consent procedure, patients will not receive treatment in another clinic while waiting for day-treatment care. After the waiting period they will be randomized into two intervention groups.

The inclusion and exclusion criteria are listed in Table 1. The number of excluded patients and the reasons for exclusion will be recorded. The diagnostic criteria are based on the DSM-V system. Initial assessment will be conducted with independent researchers who will not be involved in the further interventions. It will include a pharmacological consultation by a psychiatrist, personality diagnosis with SCID-II [24, 25], and diagnosis of clinical disorders with the Mini International Neuropsychiatric Interview 5^1^ (MINI) [71, 51]. Diagnosis and evaluation for treatment will be carried out during ambulatory sessions. The focus of the treatment is on psychotherapy, but medication will be continued or initiated if necessary during the screening procedure at least four weeks before the beginning of group psychotherapy (stable medical treatments with selective serotonin re-uptake inhibitors or serotonin-norepinephrine reuptake inhibitors, without dose changes during psychotherapy unless necessary). Baseline measurements and final adjustments of pharmacological treatment will be conducted one day before the beginning of therapy. Randomization will take place during the last diagnostic session. Participants will be randomized using random number tables. A recruitment flow chart is presented in Fig. 1. Patients are planned to be enrolled in the study for over a period of three years. Before participating in the study, each patient will be provided with written information about the study and will be invited to give written consent for inclusion. Participants who do not meet the inclusion criteria will be offered suitable alternative treatment options.

**Table 1:** Inclusion and exclusion criteria

Inclusion criteria	Current diagnosis of Axis I anxiety disorder (based on the MINI)
	Diagnosis of comorbid personality disorder (based on the SCID II) or depressive disorder (based on the MINI)
	Aged 18 to 65-years-old
	Sufficient knowledge of the Polish language
	Written informed consent from the patient
Exclusion criteria	Organic brain disorder
	Acute substance-related disorders
	Schizophrenia or bipolar affective disorder
	Severe depression (defined as over 26 points on the Beck Questionnaire)
	Cluster A personality disorders
	Current suicidal ideation and a high risk of suicide
	Restricted intellectual capacity
	Serious unstable medical problems or complications
	Concurrent psychotherapeutic treatment

### Interventions

Interventions will be offered in a daily unit setting, which is a part of the Wola Center for Mental Health at the district Wola Hospital in Warsaw. Psychodynamic and cognitive-behavioral group psychotherapy has been provided at the Wola Center since 2011. This method was developed by the current team during the last ten years in another clinical setting. When the research project starts, each group therapy will be conducted by two therapists trained in accredited cognitive-behavioral or psychodynamic/psychoanalytic therapy programs.

As is stated in the informed consent, patients will be encouraged not to commence any other therapy in the six-month period after the treatment. In cases of acute deterioration, crisis intervention will be offered. Due to ethical reasons we decided not to ask for postponing other therapy when it seems to be needed. Further treatment choices of the patients will not be controlled.

### Short-term intensive psychodynamic group therapy versus cognitive-behavioral group therapy

Both modality therapists should have at least three years’ experience in working with groups. Comprehensive treatment manuals prepared by a group of current and past members of the department’s therapeutic team, who were personally involved in the study, will be used for both treatments. The two treatments will be delivered at the same levels of frequency, defined by the total number of sessions over the study period. The number of spoken group therapy sessions lasting 90 minutes for each treatment modality will total 70 to 80 hours over a period of 12 weeks. This is the maximal period of time that can be reimbursed by insurance companies. Both approaches will contain additional interventions: psycho-drawing (one session per week), movie therapy (one session per week), and relaxation training (one session per week; see the treatment weekly schedule in Table 2). They are parts of both manuals and are aimed to intensify the therapeutic processes.

**Table 2:** Treatment weekly schedule

Time^a^	Monday	Tuesday	Wednesday	Thursday	Friday
9:00-10:30	Psychotherapy	Relaxation	Movie therapy	Psycho-drawing	Psychotherapy
	1 hour 30 minutes	1 hour 30 minutes	1 hour 45 minutes	1 hour 30 minutes	1 hour 30 minutes
10:30-11:15	Meal break	Meal break	Meal break	Meal break	Meal break
	45 minutes	45 minutes	45 minutes	45 minutes	45 minutes
11:15-12:45	Psychotherapy	Psychotherapy	Movie therapy	Psychotherapy	Psychotherapy
	1 hour 30 minutes	1 hour 30 minutes	1 hour	1 hour 30 minutes	1 hour 30 minutes
13:00-13:50	50-minute psychiatrist group consultations				

^a^Patients come to the hospital between 8 and 9 am and leave between 1 and 2 pm every day

Treatment will start in the morning hours and end in the afternoon. According to the Polish health care regulations, patients treated in a day hospital setting should have a therapeutic program for at least five hours a day. This study will be conducted in closed-ended groups. The hospital will provide a small meal during the 45-minute break between the two 90-minute psychotherapy sessions. The acceptable limit of absences is five days throughout the whole treatment. Psychotherapists will receive weekly supervision by certified supervisors. All spoken psychotherapy sessions will be recorded, and a random selection of 10 % from each therapy will be analyzed by two different independent raters on protocol adherence. This will ensure that the essential ingredients are being covered by the therapist and, if not, then the reason for the deviation will be recorded. The reported deviation will be considered minor or major by the principal investigator, who will decide if the particular group should be excluded from the study. The Comparative Psychotherapy Process Scale (CPPS) [34] will be used for this purpose, together with *ad-hoc* checklists based on specific group treatment manuals. The CPPS is designed to assess the distinctive features of psychodynamic-interpersonal and cognitive-behavioral treatments.

#### Intervention: short-term intensive psychodynamic group therapy

The form of short-term intensive psychodynamic group therapy to be used in this trial has been manualized by the research team for the study. The manual describes the aims of the treatment, specifies the therapist’s tasks and purposes, presents the therapist’s interventions, and outlines the kinds of problems and conflicts that can be found in this patient group. The manual also provides examples of session situations that are typically encountered in a day hospital setting. The manual is based on the team’s experience and on some elements of available manuals. It is rooted in the group analysis tradition [59], and is based on the group analytic psychotherapy manual as developed by Steinar Lorentzen [47]. The manual was adapted for the day treatment setting and also draws on Polish psychoanalytical group experiences as practiced in public service [58–60, 81]. This approach was initiated in the 1960s and has evolved since then.

The intervention shares the five basic assumptions of the psychodynamic approach formulated by Rutan [68]. The first is psychological determinism: the assumption that our actions are more or less determined, like our way of perceiving and recognizing. The second is unconscious processes, namely that predetermined influences may to a great degree be unconscious. The third is dynamic and goal-directed behavior: according to the dual-instinct theory, a person’s behavior serves to protect the individual from perceived danger or pain, whereas according to the object-relation theory, the behavior of a person is directed to reach a relationship and attachment. Psychology of the self claims that the goal is to formulate and strengthen the integrated self. The fourth is epigenetic development: the unconscious life of the individual originates in relation to interpersonal experiences remaining from before. People are unconsciously forced to repeat those experiences which are connected with a majority of conflicts and painful experience. Flaws in early development can be repaired if they are relived and effectively re-experienced correctively in the here and now of the transference. Finally, the assumption that different functions of mind are at work at any time, and distinct functions (id, ego, superego) may be in conflict with one another. In this approach, all anxiety disorder symptoms are understood as an expression of unacceptable unconscious fantasies and conflicts. This approach also shares the assumption that the current conflict, which is activated under the influence of current life events, is a reflection of a conflict of the past [48]. Due to the short form of the therapy, its goals are formulated realistically by taking into account the symptoms of personality disorders.

The main therapeutic factors are: 1) mirroring, namely that patients can recognize their own aspects in the fantasies, behaviors, and problems of other group members; 2) sharing, namely that patients share information, which helps them to understand themselves, their interactions with others, and their own problems; and 3) condenser phenomena, namely that unconscious material is easily expressed through the common influence of group members on one another. Shared associations help to understand the symbolic significance of experiences. Additionally, universal group factors are taken into account [87]. The therapists use techniques of clarification, confrontation, and interpretation. As in other psychodynamic short-term therapies, this approach is more structured. To intensify the therapeutic processes, three additional techniques are used: 1) memoir, namely that each patient describes his or her own life story, which consists of important emotional experiences, and reads it during one therapeutic session; 2) elements of role playing and/or psychodrama, namely that the patient presents his or her way of talking to important persons; 3) a summary, namely that patients receive written structured feedback from other patients in the final stage of therapy.

Application of these techniques results from the need for patients to concentrate on the therapeutic goals and for greater structuralization in patients with personality disorders. It is characteristic of this type of therapy to observe and discuss the manner of how a patient interacts with other patients in a group and with the therapist (transference), and afterwards to relate this to the patient’s functioning in past relations and in other relations beyond the therapy. A significant factor is exploring and modifying immature defense mechanisms, with a major focus on the mechanisms of projection and acting-out, and on increasing the employment of mature defense mechanisms. The group process is of great importance; it is assumed to have both a conscious and unconscious aspect. The course of therapy consists of phases that are supposed to stimulate various conflicts in different patients. The therapists are more active here than in the classical form of group analysis: they employ interventions more often, such as explaining and naming reality, and are less focused on patients’ fantasies. In classic group analysis, the group therapist does not function as a group leader but as a group conductor, frustrating regressive needs and thus replacing the leader’s authority by that of the group: all members interpret, analyze, and support one another, including the conductor [43].

#### Intervention: short-term intensive transdiagnostic cognitive-behavioral group therapy

The short-term intensive transdiagnostic cognitive-behavioral group therapy (GCBT) was designed to conduct treatment in naturalistic settings of diagnostically mixed anxiety disorder patients with comorbid personality disorders [36]. It is based on the transdiagnostic approach to emotional disorders, and particularly anxiety disorders, which has been in development for over the past 10 years [4, 5, 55]. Additionally, it includes interventions for personality disorders based on schema therapy and modes of personality disorders. This approach assumes high overlap between anxiety disorders and is focused on their common aspects. Preliminary data indicate that GCBT is equally effective in the treatment of individuals with different anxiety disorders [56].

The short-term intensive GCBT that has been developed by our group shares the classical assumptions of CBT [6], namely that the emergence and persistence of psychopathological symptoms results from maladaptive thinking styles and cognitive bias (the cognitive model), and maladaptive behavioral strategies (the behavioral model). Anxiety disorders in particular are described as resulting from distorted beliefs focused on threat (physical or psychological) and an elevated sense of personal vulnerability [9]. Therefore, short-term intensive GCBT, just like standard CBT, is focused on helping patients identify and modify distorted cognitions, and on using techniques based on learning principles, mainly exposure, to help them eliminate anxiety.

Short-term intensive GCBT, similarly to most CBT therapies, is task-oriented and designed for finding and solving problems. Among the therapeutic techniques there are: monitoring and identification of negative automatic thoughts and related emotional experiences, physical sensations and behaviors (in the form of diaries), various techniques for restructuring and challenging dysfunctional thinking, creating a hierarchy of anxiety-provoking situations, performing exposure (imaginal and *in vivo*), behavioral experiments, relaxation techniques, developing goal-setting and problem-solving skills, and social skills training [41]. What differentiates GCBT from CBT is that the strength of group cohesion is employed, that is, a sense of belonging and confidence in the group’s members and believing that its goals are important [77, 87]. GCBT creates a good environment for such phenomena as modeling, social learning, and exposure. Participating in the group gives the patient the opportunity to employ characteristic group therapeutic factors, such as hope, universality, information sharing, altruism, corrective recapitulation of primary family experience, social skills development, and imitation [87].

The GCBT therapy process is divided into modules that reflect the phases and stages of the therapeutic process (P Holas and H Suszek, unpublished work). A weekly schedule includes cognitive therapy and behavioral therapy (exposure, *in vivo*) sessions as well as social skills training, including mainly assertiveness. As a result of such a schedule, each week consists of four elements: a homework discussion (weekend task), the presentation of new material during psychoeducation and therapy sessions, training new skills (examples from the lives of patients, role play, and exposure), and planning the next homework assignment. As a result, patients learn new skills, practice them in a safe environment, and then gradually introduce them into their lives outside the group.

### Assessments

Patients are subjected to four assessments: 1) at the screening; 2) at baseline after randomization; 3) at the end of the 12-week treatment; and 4) six months after the end of therapy in order to evaluate the longer-term effects of the treatments. Patients in the wait-list group will have one additional assessment at the end of the 12-week waiting period.

Information from the screening will be used to perform stratified randomization and to validate the inclusion and exclusion criteria. Patients will be assessed by independent and trained clinicians who will remain blind to the treatment conditions, and who will not be involved in direct clinical care of any of the patients. Primary outcome measures and secondary outcome measures can be distinguished in this study. The primary outcome measures include self-report symptoms of anxiety, observer-rated symptoms of anxiety, global improvement, and dichotomized diagnosis of anxiety disorder (improved or unimproved). The secondary outcome measures include personality, depression, self-esteem, defense mechanisms, beliefs about self and others, interpersonal problems, object relations, parental bonding, meta-cognition, and quality of life. The following sociodemographic data will be collected: sex, age, marital status, education, past treatments, symptom history, and medication. We will also examine patients’ expectancy toward change and treatment. Some of the variables, such as the presence or absence of a personality disorder, will be treated in exploratory analyses as predictors and moderators of treatment response. The time points of assessment and measures are presented in Table 3.

**Table 3:** Instruments at different assessment points

Instrument	Screening	Baseline	End of Treatment	6-Month Follow-up
STAI		X	X	X
HAM-A		X	X	X
CGI-S		X	X	X
CGI-I		X	X	X
M.I.N.I.	X		X	X
SCID-II	X			
BDI-II	X		X	X
OQ-45		X	X	X
SES		X	X	X
DSQ-40		X	X	X
PBQ		X	X	X
IIP		X	X	X
DWM-S		X	X	X
ECR		X	X	X
BORRTI		X	X	X
MCQ		X	X	X
SWLS		X	X	X
CEQ		X		

STAI: State-trait anxiety inventory; HAMA-A: Hamilton anxiety rating scale; CGI-S: Clinical global impression - severity scale; CGI-I: Clinical global impression - improvement scale; M.I.N.I.: Mini-international neuropsychiatric interview 5; SCID II: Structured clinical interview for DSM-IV personality disorders questionnaire; BDI-II: Becks depression inventory II; OQ-45: Outcome questionnaire; SES: Self-esteem scale; DSQ-40: Defense style questionnaire; PBQ: Personality beliefs questionnaire; IIP: Inventory of interpersonal problems; DWM-S: Dysfunctional working models scale; ECR: Experiences in close relationships; BORRTI: Bell object relations and reality testing inventory; MCQ: Meta-cognitions questionnaire; SWLS: Satisfaction with life scale. CEQ: Credibility/Expectancy questionnaire

### Instruments

#### Primary measures

The following instruments will be used:

1. State-Trait Anxiety Inventory (STAI) [72, 86]. The STAI measures a person’s situational (or state) anxiety, as well as the amount of anxiety a person generally feels most of the time (trait). The two self-report scales contain 20 items each.
2. Hamilton Anxiety Rating Scale (HAM-A) [33]. The HAM-A is an interviewer-administered and rated measure of the severity of anxiety symptoms. The scale consists of 14 items, each defined by a series of symptoms, and measures both psychic anxiety (mental agitation and psychological distress) and somatic anxiety (physical complaints related to anxiety).
3. Clinical Global Impression - Severity scale (CGI-S) [32]. The CGI-S is a seven-point scale which requires that the clinician rate the severity of the patient’s illness at the time of assessment, relative to the clinician’s past experience with patients who have the same diagnosis.
4. Clinical Global Impression - Improvement scale (CGI-I) [32]. The CGI-I is a seven-point scale which requires that the clinician assess how much the patient’s illness has improved or worsened, relative to a baseline state at the beginning of the intervention.
5. Mini-International Neuropsychiatric Interview 5 (MINI) [51, 71]. The MINI is a short diagnostic interview based on the DSM-IV criteria, which focus on the existence of current psychiatric disorders. It consists of separate modules to diagnose specific disorders.

#### Secondary measures

The following instruments will be used:

1. Structured Clinical Interview for DSM-IV Personality Disorders Questionnaire (SCID-II) [24, 25]. The SCID-II is an observer-based diagnostic interview used to determine DSM-IV Axis II disorders.
2. Becks Depression Inventory II (BDI-II) [8]. The BDI-II is a 21-item self-report inventory used to assess DSM-IV depressive symptoms. Each item consists of four statements indicating increasing symptom severity.
3. Outcome Questionnaire (OQ-45) [28, 45]. The OQ-45 consists of 45 items and is designed to evaluate a broad range of psychological problems and symptoms of psychopathology. It contains three subscales: 1) symptom distress, 2) interpersonal relations; and 3) social role.
4. Self-Esteem Scale (SES) [22, 67]. The SES is a 10-item self-report scale that measures global self-worth by measuring both positive and negative feelings about the self.
5. Defense Style Questionnaire (DSQ-40) [1, 13]. The DSQ-40 is a 40-item self-report questionnaire designed to measure an individual’s propensity towards three various defense styles (mature, neurotic, and immature), along with 20 individual defense mechanisms.
6. Personality Beliefs Questionnaire (PBQ) [7]. The PBQ is a 126-item self-report measure of beliefs associated with 10 DSM-III-R and DSM-IV personality disorders (represented as subscales): 1) avoidant; 2) dependent; 3) obsessive-compulsive; 4) histrionic; 5) passive-aggressive; 6) narcissistic; 7) paranoid; 8) schizoid; 9) antisocial, and 10) borderline.
7. Inventory of Interpersonal Problems (IIP) [37]. The IIP is a 64-item self-report questionnaire that inventories the interpersonal problems that people experience. It contains eight scales that assess different aspects of an individual’s interpersonal difficulties: 1) domineering/controlling; 2) vindictive/self-centered; 3) cold/distant; 4) socially inhibited; 5) non-assertive; 6) overly accommodating; 7) self-sacrificing; and 8) intrusive/needy.
8. Dysfunctional Working Models Scale (DWM-S) [54, 62]. The DWM-S is a 35-item self-report measure of dysfunctional working models of the self and others.
9. Experiences in Close Relationships (ECR) [14, 74]. The ECR is a 36-item self-report measure of adult attachment. It groups people into four different categories on the basis of scores along two scales: avoidance and anxiety.
10. Bell Object Relations and Reality Testing Inventory (BORRTI) [10, 73]. The BORRTI is a 45-item self-report measure designed to evaluate the ability to sustain essential relationships and to accurately identify internal and external reality.
11. Meta-Cognitions Questionnaire (MCQ) [17, 29]. The MCQ is a 65-item self-report scale developed to assess several dimensions of metacognition that is thought to be relevant to psychopathology. Its five subscales are: 1) positive beliefs about worry; 2) negative beliefs about thoughts concerning uncontrollability and danger; 3) cognitive confidence (assessing confidence in attention and memory); 4) negative beliefs concerning the consequences of not controlling thoughts, and 5) cognitive self-consciousness (the tendency to focus attention on thought processes).
12. Satisfaction With Life Scale (SWLS) [20, 41]. The SWLS is a five-item self-report global measure of life satisfaction.
13. Credibility/Expectancy Questionnaire (CEQ) [18]. The CEQ is used to measure expectancy for change and treatment credibility as potential confounds for outcome. It is comprised of six questions.

### Record retention and confidentiality

Epidemiological, clinical, and all outcome data from each patient will be recorded in an electronic database by a clinician responsible for the study. All records will be stored for 10 years from the date of the last study publication. All computer databases will include a unique participant identifier and not the participant’s name and address. Consent forms and other documents, including the participant’s name, will be stored separately from the questionnaires and other trial documents in locked cabinets.

### Withdrawal from the trial

Participants may voluntarily withdraw from the trial for any reason. A patient’s withdrawal from the trial will not affect that patient’s access to treatment.

### Sample size calculation

The sample size calculation was based on previous trials showing the effectiveness of group CBT or psychodynamic group therapy in anxiety and personality disorders; however, very few studies of this kind have been conducted. Additionally, we decided to base our study on trials showing the effectiveness of group psychotherapy in general, and on trials showing the effectiveness of group psychotherapy in day hospitals.

Burlingame *et al*. [15] estimated the effectiveness of group psychotherapy in a meta-analysis of 111 experimental and quasi-experimental studies that were published over the past 20 years. Based on 51 studies which compared active group treatment versus a wait-list control, the reported average effect size was 0.58. Effect sizes for comparisons with a wait-list control for different diagnoses and therapeutic orientations were not reported. Petrocelli [62] employed a meta-analysis to examine the effectiveness of GCBT based on 12 studies, in which GCBT was compared with no treatment. The average weighted mean effect size for GCBT versus no treatment was 0.59. Wersebe *et al*. [82] conducted a meta-analysis for the effectiveness of GCBT for social anxiety disorder. The inclusion of 11 RCTs comparing interventions with control conditions showed a pooled effect size of 0.53 in favour of the interventions. A review conducted by the Centre for Psychological Services Research at the University of Sheffield to assess evidence for the efficacy and effectiveness of psychodynamic group therapy (including group analysis) identified 37 studies [11, 12]. Only one study included the wait-list control [78], but was limited to binge-eating disorder patients. Knijnik *et al*. [44] conducted an RCT which showed that patients with generalized social phobia treated with psychodynamic group therapy improved significantly on their social anxiety symptoms as compared to the placebo group, showing an effect size of 0.83. The most similar trials to ours are two RCTs reported in a Cochrane review which deal with group psychotherapy as a component of day treatment [2, 50, 63]. The first study found that day care patients suffering from affective disorders and personality disorders experienced a significant improvement as compared to patients in the wait-list control group [63]. The mean effect size for 17 outcome variables was 0.71. In the second study, day care treatment (18-week group psychodynamic and cognitive-behavioral psychotherapy) was compared to individual psychotherapy in patients with personality disorders [2]. No significant differences in improvement were observed between the two groups.

The effects of the intervention on symptoms of anxiety are the primary outcome measures in our study. Although the study’s general aim is to compare both treatments with the wait list, an additional comparison has been planned of both treatments on the outcomes. Comparison between all three groups is possible for two subsequent measurements. Due to ethical reasons, subjects from the wait list will start therapy soon after the second measurements and will not be available to participate in the study during the third measurement. As a result, that data will be analyzed in a 3 × 2 experimental plan (three groups available for two repeated measures) and in a 2 × 3 experimental plan (two groups available for three repeated measures). Based on the trials mentioned above, we assumed that we would be able to show the differences between the interventions and the control conditions with a standardized effect size of 0.58 (the lowest value effect size presented in the literature) for continuous outcomes. As this effect size was Cohen’s d suited for t-test comparisons, we conducted a conversion into f suited for analysis of variance (f = 0.29). The sample size was calculated using the G*power software program [23]. Assuming an alpha of 0.05, with a power (1-β) of 0.80, a minimum number of 96 patients is required for a 2 × 3 experimental plan, and a minimum number of 120 patients is required for a 3 × 2 experimental plan. Furthermore, for the purpose of being able to identify the difference between the two treatments, we followed the design of the most similar study [2] and assumed effect sizes of 0.24 for continuous outcomes, which leads to a total sample size of 171 participants for a 3 × 2 experimental plan and a total sample size of 140 participants for a 2 × 3 experimental plan. We will also test possible within-between group interaction, however its effect size is not available in literature. We assumed a medium effect size for the interaction (f = 0.25), which needs 156 participants in a 2 × 3 plan and 159 participants in a 3 × 2 plan. Assuming that there is a dropout rate of 25 %, this means that 199 participants will need to be recruited.

### Statistical analysis

All data analyses will be performed using SPSS Statistics software v21.0 (IBM Corporation, Armonk, NY, USA). All randomized patients will be analyzed, including those who stop receiving treatment, on an intention-to-treat (ITT) as well as treatment-completer basis. The multiple imputation method will be used for missing data when appropriate. Analyses of variance with repeated measures (three measurement points: pre, post, and follow-up) will be applied for continuous data (primary outcomes: STAI, HAM-A, CGI-S, and CGI-I; secondary outcomes: BDI-II, OQ-45, SES, DSQ-40, PBQ, IIP, DWM-S, ECR, BORRTI; MCQ, and SWLS). Primary planned comparison is the interaction between the time (pre-to-post and post-to-follow-up change on anxiety) and group variables (CBT versus wait list; PDT versus wait list). Chi-squared tests will be applied for dichotomous data (MINI). Additionally, random effects models for repeated measures will be used. Multiple regression analyses will be applied to test moderators of change that is to show whether each of the interventions is more effective for some groups of patients (interventions, diagnoses, combination of diagnoses and baseline measurements as predictors). Multiple regression analyses will be also applied to search for mediators of change that are theory-specific (for example, changes in beliefs for CBT and changes in defense mechanisms for psychodynamic therapy). We will take into account to what extent the patients were exposed to the intervention by analyzing the dose-effect relationship.

## Discussion

This study examines whether day treatment based on short-term intensive group therapy is effective. A further objective is to examine which of the two therapy modalities, psychodynamic or cognitive-behavioral, is more effective. We also plan to examine the predictors, moderators, and mediators of therapeutic change. The current study has several strengths.

Despite a large number of RCT studies showing the advantages of using psychodynamic therapy and CBT for anxiety disorders and personality disorders [35, 70], there is still insufficient evidence for the efficacy of the group forms of these approaches, especially the psychodynamic orientation. Most studies show that the efficacy of treating anxiety disorders with cognitive-behavioral group therapy is comparable to its individual form [31, 53, 66, 69, 84]. There is still a lack of similar group versus individual comparisons for the psychodynamic approach [15].

A meta-analysis conducted by Tolin [79] that examined the effectiveness of CBT and other therapies showed the superiority of CBT over alternative therapies among patients with anxiety or depressive disorders. A recent meta-analysis by Baardseth *et al*. [3] showed no differences between CBT and non-CBT treatments for anxiety disorders. In those analyses, however, almost none of the trials were completed in community settings.

Group psychotherapy as day treatment differs from outpatient therapy in intensity (usually five sessions per week versus one session per week). To date, there have been only two RCTs [2, 63] comparing psychodynamic group psychotherapy and cognitive-behavioral group psychotherapy as components of day treatment with outpatient therapy. However, neither of the studies addressed anxiety disorders.

There have been several studies about the efficacy of day treatment of anxiety disorders in Poland [13, 49, 75, 83, 85]. These studies show promising results, however, none of them were RCTs. This will be the first RCT to evaluate manualized psychodynamic group therapy and cognitive behavioral group therapy in a day treatment setting for people with anxiety disorders. The design of the study overcomes the disadvantages of previous studies in that it provides a randomized controlled design, consists of a large sample size, adequate inclusion criteria, an adequate treatment protocol, and a clear separation of intervention conditions.

Another strong aspect of the study is its external validity, since the comorbidity of anxiety disorders with depressive and/or personality disorders is controlled. Furthermore, the results may be highly generalizable as the intervention is studied in its natural setting, and recruitment strategies of both the study and the day hospitals are very similar. The study combines the elements of RCTs and naturalistic studies. The study results may be of great relevance to the health care policy, health insurance companies, and boards of psychotherapists, and may contribute to improving the quality of treatment and to a reduction in health care costs. This issue is relevant to the Polish health care system, in which evidence-based practice standards are becoming more important and institutionally acknowledged. Another strong point of this study is the broad array of outcome estimates, which will allow us to measure the effects of treatment in many areas of life.

Many variables and outcome measures may also be tested as moderators of change, such as a particular anxiety disorder or a particular combination of diagnoses, self-concept, and parental bonding. Furthermore, by having a broad range of outcome measures, our study will offer an opportunity to look at the mediators of change that are theory-specific (such as changes in beliefs for CBT and changes in defense mechanisms for psychodynamic therapy). It would be interesting to test whether there are shared mechanisms of change.

We recognize a number of limitations in this study and suggest improvements for future research. First, generalizing the research findings will be limited because the trial will be a single-center RCT; future multi-center trials are needed. Second, having a passive wait-list control group does not offer the best control for the effects of expectancy. By including the condition of a placebo control in which no intervention is given but only the support of a therapist (support group), we would be able to examine the non-specific effects of group interventions, such as social cohesion and expectation of gain. An active control group is not possible because patients would have to take three months off of work and this period would not be covered by their insurance. Another limitation of this study concerns the use of self-report instruments as outcome measures.

The last two limitations are related to the complexity of the interventions and treatment setting. Both treatments consist of many elements, of which verbal psychotherapy is only one. Therefore, it is not possible to tease out whether potential changes can be attributed to psychotherapy or to other aspects of the interventions. We also do not control the exact time that patients will show up every day at the hospital before therapy, and the time when they leave the ward after therapy. This limitation is connected with the formal requirements of the Polish health care system.

## Trial status

Trial recruitment is anticipated to begin in September 2015.

## Endnotes

^1^SCID criteria for personality disorders and MINI criteria for anxiety disorders will be additionally verified with the DSM-V.

## Abbreviations

BDI-II: Becks depression inventory II
BORRTI: Bell object relations and reality testing inventory
CBT: Cognitive-behavioral therapy
CEQ: Credibility/Expectancy questionnaire
CGI-I: Clinical global impression - improvement scale
CGI-S: Clinical global impression - severity scale
CPPS: Comparative psychotherapy process scale
DSM-IV: Diagnostic statistical manual 4th edition
DSM-5: Diagnostic statistical manual 5th edition
DSQ-40: Defense style questionnaire
DWM-S: Dysfunctional working models scale
ECR: Experiences in close relationships
HAMA-A: Hamilton anxiety rating scale
ICD-10: International classification of diseases
IIP: Inventory of interpersonal problems
MCQ: Meta-cognitions questionnaire
MINI: Mini-international neuropsychiatric interview 5
OQ-45: Outcome questionnaire
PBQ: Personality beliefs questionnaire
RCT: Randomized controlled trial
SCID: Structured clinical interview for DSM-IV personality disorders questionnaire
SCID II: Structured clinical interview for DSM-IV personality disorders questionnaire
SES: Self-esteem scale
STAI: State-trait anxiety inventory
SWLS: Satisfaction with life scale
